# Students’ Physical Activity Profiles According to Children’s Age and Parental Educational Level

**DOI:** 10.3390/children8060516

**Published:** 2021-06-18

**Authors:** Inés M. Muñoz-Galiano, Jonathan D. Connor, Miguel A. Gómez-Ruano, Gema Torres-Luque

**Affiliations:** 1 Department of Pedagogy, Faculty of Humanities and Education Science, University of Jaén, 23071 Jaén, Spain; imunoz@ujaen.es; 2 Department of Sport and Exercise Science, James Cook University, Townsville, Douglas QLD 4811, Australia; jonathan.connor@jcu.edu.au (J.D.C.); miguelangel.gomez.ruano@upm.es (M.A.G.-R.); 3 Department of Social Sciences of Physical Activity, Sports and Leisure, Technical University of Madrid, 28040 Madrid, Spain; 4 Department of Plastic, Music and Corporal Expression, Faculty of Humanities and Education Science, University of Jaén, 23071 Jaén, Spain

**Keywords:** children, behavior sedentary, cluster, education, physical activity

## Abstract

The aim of this study was to identify different profiles of physical activity (PA) behaviors according to the school student’s age stage and their parents’ or guardians education level. Seven hundred twenty-seven students and parents of different educational stages were invited to take part in this study. The participants included, Preschool (1 to 5 years old), Primary School (6 to 11 years old), Secondary School (12 to 15 years old), and High School (16 to 18 years old). A questionnaire to assess the educational level of parents (low, intermediate, and high) and their child’s PA level and sedentary behaviors across various age stages was administered. The results showed a number of different physical activity profiles for preschool (4), primary (6), secondary (7) and high school (2) students. Primary and secondary school children’s behavioral profiles were reported to differ significantly between both physical activity levels and sedentary behaviors, while preschool students’ behavioral profiles only differed between sedentary behaviors. Higher parental education was most prevalent in clusters with significantly higher levels of PA in primary and secondary students, while there were equivocal trends for parental education level influencing behavioral profiles of high school students. These findings suggest there is some association between the behavioral profiles of student’s physical activity and sedentary behavior, and parental education level, most noticeably during the early to middle age stages.

## 1. Introduction

Life style can determine the quality of life and health of an individual from childhood [1]. Globally, changes in lifestyle and leisure habits that lead to an increase in sedentary behavior are being promoted [2,3,4,5,6], and increasing physical inactivity [7,8,9,10] is becoming a major risk factor for health from an early age [4,5,7,11,12,13].

High levels of sedentary lifestyle and decreased physical activity (PA) are related to public health problems [1,14] including overweight and obesity [6,15,16,17], increased cardiometabolic risk [18,19] and detrimental effects on psychosocial well-being [3,20]. However, sedentary behaviors (behaviors with low energy expenditure) are becoming increasingly predominant in schoolchildren between the ages of 5–17 [7,21,22] despite the well-known recommendations of moderate to vigorous PA per day (i.e., 60 min).

Although there have been unequivocal findings when examining the association between levels of PA and different sedentary behaviors, the latter is relevant to investigations on PA due to its prejudice towards health, and necessitating preventive measures to help promote an active and healthy lifestyle [7,16,23]. The quality of life and health in adulthood generally depends on the habits adopted from the adolescent years [24,25]. In this sense, it is vital to establish positive behaviors from the early stages of life, since the habits acquired at an early age tend to be maintained over time [25].

To increase PA and decrease sedentary lifestyle in schoolchildren, more information about behaviors in the home environment is needed [26,27,28,29,30]. Various studies indicate a decrease in levels of PA and an increase in sedentary behavior as individuals age [6,31,32,33]. Furthermore, sedentary behavior often differs according to age and gender [6,16,20,34,35]. Girls have been shown to consume more screen time and engage in less PA compared to boys [7,22]. International Children’s Accelerometry Database indicates that boys tend to spend more time engaged in PA (up to 70%), with a higher sedentary lifestyle reported for girls; a difference that is constantly increasing from childhood to adolescence [12].

Recent studies have shown the parents’ educational level may be an influencing factor of PA levels in children, although it has been investigated less frequently [14,36,37]. The data have suggested that parents with a higher degree of education have more active [30,37] and less sedentary children [5]. The parents’ educational level is found as a relevant factor for the socioeconomic status, and may explain differences in the physical and social environment in the home of physically active children [38]. Thus, children from families of a low socio-economic level tend to report spending more time in front of the screens than those in a family from a higher socioeconomic status [1,5,39]. In contrast, those students with a high socio-economic level, have a healthier lifestyle [40,41] and participate more frequently in PA [14].

Therefore, combining what happens throughout the school stage in terms of the practice of PA and sedentary time, can contribute to generating greater healthy lifestyle promotion strategies. The aim of this study was to identify different profiles of PA behavior related to children’s age and parents’ education level.

## 2. Materials and Methods

### 2.1. Sample

Seven hundred twenty-seven students and parents of different educational stages were invited to take part in this study. The participants included, Preschool (1 to 5 years old) (*n* = 179; 4.08 ± 0.83 years; 107.0 ± 8.97 cm; 18.8 ± 4.14 kg; 16.6 ± 3.19 kg/m^2^), Primary School (6 to 11 years old) (*n* = 284; 9.37 ± 1.35 years; 143.2 ± 8.97 cm; 38.9 ± 11.2 kg; 18.7 ± 3.80 kg/m^2^), Secondary School (12 to 15 years old) (*n* = 230; 13.1 ± 0.94 years; 143.3 ± 9.22 cm; 54.6 ± 10.7 kg; 20.1 ± 5.07 kg/m^2^), and High School (16 to 18 years old) (*n* = 34; 16.1 ± 0.23 years; 172.1 ± 7.55 cm; 65.7 ± 13.7 kg; 22.0 ± 3.88 kg/m^2^) students. The inclusion criteria for taking part in the study were: (a) students enrolled in Preschool, Primary, Secondary or High-school Education; and (b) not have health problems that limits or prevent being part of a physical activity. In an initial stage, the educational centers as well as the parents and/or guardians were informed of the aims of this study. All parents or guardians provided written informed consent to participate. This study obtained the approval of the Ethics Committee of the local institution (University of Jaén, Jaén, Spain, JUN.17/6).

### 2.2. Procedures

#### 2.2.1. Educational Level of Parents/Guardians

The parents/guardians’ educational level was obtained through a questionnaire, where they indicated the highest level of education they had attained based on the Spanish education system. Accordingly, the response options were pooled into three levels: (a) Low level: including no graduation, Primary/EGB, Secondary/ESO; (b) Medium level: including Vocational Training I, Middle level training cycles, Baccalaureate/BUP/COU, Professional Training II, Higher degree training cycle; and, (c) High level: including a University degree, technical engineering or higher engineering graduate, Masters, or Doctorate graduate. The questionnaire was completed in person. The questionnaire was completed by parents or, by the children themselves if they are of the age of eight or older. These educational levels were used in accordance with previous studies employing this method [5].

#### 2.2.2. Physical Activity Practice

Information regarding the PA practice was gathered by utilizing a series of questions about (1) children’s active displacement practices (i.e., travelling to and from school), (2) unstructured PA practices, and (3) structured PA practices. The questionnaire was completed in person. The questionnaire was completed by parents or by the children themselves if they are of the age of eight or older. Active movement information was obtained via qualitative-style questions about how children travel to and from school each day (e.g., (walking, cycling, car, motorcycle, bus). A follow up quantitative-based question involved obtaining the amount (in minutes) of PA during a round trip to and from school (less than 10 min, between 10–15 min, between 15–20 min, between 20–30 min, more than 30 min). Importantly, volume of PA was only included in the calculation of total activity profile (PA) when an active displacement (e.g., walk, cycle) was selected.

Unstructured PA practice was obtained via questions regarding children’s play time in the backyard, in the park, playground, or other opportunities for PA, and where PA practice (i.e., volume played) differentiating the week and at the weekend. Structured PA practice was obtained via questions about children’s time spent engaged in extracurricular sporting (citing all sports), and physical activities (other possible activities). Additionally, the time spent during the week was compared with the time spent at the end of week. The total hours of PA were added once the questionnaire was fulfilled, differentiating between weekdays (Monday to Friday), and weekend (Saturday and Sunday). The questionnaire used questions based on several previous studies [29,42,43].

#### 2.2.3. Sedentary Behavior

Sedentary behavior was determined through the Health Behavior in School-aged Children (HBSC) questionnaire [44]. Preschool students’ parents answered the questions. The questionnaire included six items showing the number of daily hours spent watching television across the week (Time watching TV; Time using tablet/similar; Time doing homework, all in weekdays and weekend). Each of the questions included 9 possible responses, including: 1 = no time, 2 = half an hour, 3 = one hour, 4 = two hours, 5 = three hours, 6 = four hours, 7 = five hours, 8 = six hours and 9 = seven hours or more. The questionnaire reliability has been previously reported as high (Cronbach’s alpha = 0.72; 0.75; 0.72 in the three dimensions respectively), and successfully adopted in previous studies [45].

### 2.3. Statistical Analysis

First, descriptive analyses were calculated for continuous and categorical variables (mean, standard deviation and frequencies). Second, a two-step cluster analysis was run to classify the students based on the PA, and parent-related variables and according to the educational stage (Low, Medium and High Level). This model establishes the best number of students’ groups (clusters) based on the Schwartz’s Bayesian Information Criterion (BIC = 783.31, 1115.26, 945.54, and 248.23). In addition, the Silhouette measure revealed good models classifying the students for infant, primary, secondary and high school (greater than 0.47). The sample was then divided into four, six, seven and two groups, respectively. Lastly, the one-way ANOVA was used to compare the PA variables among clusters in each educational stage. The Bonferroni post hoc test was applied to check pairwise comparisons. The partial eta squared effect size (ES) was used to test the impact of differences considering the following interpretation: small = 0.01; moderate = 0.06, and large = 0.14 [46]. All statistical analyses were run using the statistical software IBM SPSS for Windows, version 24.0 (IBM. Corp, Armonk, NY). The significance level was set to *p* < 0.05.

## 3. Results

Table 1 summarizes the main values for each group obtained via Two-step cluster considering the PA level and the parent’s characteristics. Based on this machine learning splitting model the students can be classified into different groups in each school age stage.

Table 1 includes the number of groups and characteristics in each of the educational stages.

The preschool category had four groups of PA behavior profiles. Group 1, has parents with high educational level, included only boys, and with the lowest values of sedentary time, which was mainly reflected in the behavior of watching TV. Group 2 included more boys than girls, with parents predominantly with a high educational level compared to medium or low educational levels, and they have, in turn, the most pronounced sedentary time. Group 3 was comprised of girls, with parents with a high educational level and spent too much time in sedentary activities, mainly watching TV. Group 4, comprised of boys and girls practically in the same proportion with parents with a medium educational level, and with times of sedentary behavior higher than group 1 and 3.

The primary school category had six PA behavioral profile groups. Group 1, are boys with parents with a low educational level, with considerable sedentary behavior, reflected in the behavior of watching TV. Group 2, are both boys and girls having parents with a high educational level, and in turn, with marked sedentary time. Group 3, are girls, with parents with low educational level and similar sedentary behavior. Group 4 are boys and girls, although boys are more present, predominantly parents with a medium educational level and a very strong presence of sedentary behavior, where time watching TV stands out. Group 5 and 6 are girls, with parents with medium and high educational level, where there are the lowest times of sedentary behavior.

The secondary school category had a total of seven PA behavioral profile groups. Group 1, are boys and girls (although more boys), parents predominantly with a medium educational level and a strong sedentary behavior reflected in time watching tablet/similar. Group 2, are Boys with parents with a high educational level and a sedentary behavior very divided between doing homework, watching TV or watching tablet/similar. Group 3, are girls with parents with a low educational level and sedentary behavior very divided between doing homework, watching TV or watching tablet/similar. Group 4 are girls, but with parents with a medium educational level and a sedentary behavior similar to those described above. Group 5, are girls with parents with a low educational level showing sedentary behavior time in line with the above. Group 6 and 7 have a similar composition, only made up of children and parents with a mainly medium educational level.

At High School, there were two identified PA behavioral profile groups. Group 1, are boys, with a high dedication to homework and where the influence of the parents’ educational level is very distributed although there is a tendency towards a high educational level. Group 2, are girls, where they spent more time dedicated to duties and with parents/guardians mainly showing a low educational level.

Table 2 shows the differences in each of the groups classified in each educational stage.

As the data show, in Preschool, there is a marked increase in sedentary behavior in group 2 than the other groups (*p* < 0.001). No statistically significant difference was found between any other group with respect to physical activity. In Primary, the total time of PA is significantly higher in groups 1, 2, 3, compared to groups 5 and 6 (*p* < 0.05). In turn, group 4, has a higher time spent in sedentary behavior in relation to the other groups (*p* < 0.001), both in time watching TV, Tablet/similar and homework (*p* < 0.001). In these last activities, there are higher times, than in groups 1,2, 3 compared to other profiles (*p* < 0.05). In Secondary, group 3 and 4 have a longer weekly PA time than all other groups (*p* <0.001), joining more PA practice in group 2 vs. group 5. And there were differences between group 5 and group 7 (*p* < 0.001). Regarding sedentary behavior, there is a greater time in group 1 and 2 compared to all the others (*p* < 0.01), which is reflected in the same way with passive behaviors including watching TV, tablet/similar and doing homework (*p* < 0.01). No difference of any kind is shown between the two High School groups.

## 4. Discussion

The aim of the current study was to identify different profiles of PA behaviors according to the children’s school age level and parents’ education level. The findings suggest that there are numerous different activity profiles for preschool, primary and secondary school children, and only a small number of activity profiles for high schoolers. Primary and secondary school children’s behavioral profiles differ significantly between PA levels and sedentary behaviors, while preschoolers only differed between sedentary behaviors. Health promotion strategies may benefit from targeting preschool to reduce their sedentary behaviors, and target both primary and secondary school children to increase their PA levels and reduce their sedentary behavior. Finally, there was also equivocal trends for parent’s education attainment influencing PA or sedentary time and behaviors between profiles of high school students.

In order to increase PA and decrease sedentary lifestyle in schoolchildren, it is critical to understand the behaviors in the home environment [26,29]. For preschool behavioral clusters, clusters 1 and 3 represented boys and girls (respectively) with highly educated parents, while cluster 4 represented an even mix of boys and girls with predominately medium educated parents, and cluster 2 represented a combination of boys and girls with a combination of various parent-education levels. Cluster 2 was also significantly higher than all other clusters for sedentary time and sedentary behaviors (including time watching TV, time using tablet/similar and time doing homework). These findings are somewhat in line with previous work reporting lower parental education levels being associated with home environments that lead to greater situations for sedentary behavior, and fewer situations for PA [47]. Tandon and colleagues [47] reported young children with highly-educated parents had significantly less screen time, and less access to media, than their lower-educated counterparts. Preschool represents a crucial age where greater PA levels and lower sedentary behavior are positively associated with future positive health outcomes, such as increased cardiorespiratory fitness [48], psychosocial wellbeing [49] and fundamental movement skill mastery [50], thus needs to be encouraged via parental engagement [51].

It is also important for primary age school children to promote enough PA situations, in order to master fundamental movement skills, and begin to develop more sport-specific movement patterns. Primary school age children in this study reported the second highest number of PA behavioral clusters across all age groups. Cluster 1 (boys) and cluster 3 (girls) represented low educated parents, while cluster 4 (mixed gender) and cluster 6 (girls) represented medium educated parents, and cluster 2 (mixed gender) and cluster 5 (girls) represented high educated parents. Particularly, boys and girls with parents with a high educational level were reported carrying out more PA (Cluster 2 and 5) than the other groups. This finding is similar to the available research [14,37,52]. Additionally, Walsh et al. [53] indicated the importance of the family in the volume of total time of PA practice of their children, although sometimes, the parents’ subjective perception does not reflect this association. Clusters 1 and 3 (low- parental education level) both reported children with significantly greater amounts of sedentary time and behaviors than clusters 5 and 6, which both represented girls and high- and medium-educated parents, respectively. Interestingly, cluster 2 represented high parental education level and a mixed gender, whilst also demonstrating greater levels of sedentary behaviors than cluster 5 and 6. It is at this age that school children begin to place greater emphasis on schoolwork, which is a key factor in sedentary behavior. Cluster 4 in particular demonstrated extraordinarily high levels of sedentary time and behaviors, including time doing homework. Additionally, it also showed low PA values. Furthermore, the relationship between parental education level or gender with sedentary behavior was not clear. While this cluster 4 represented a very small minority (4.2%), policymakers tasked with encouraging more active behaviors of young school children may need to further investigate other factors that contribute to the high sedentary behaviors.

Secondary age school children had the greatest number of PA profile clusters. Cluster 3 and 5 both represented girls with low parental education levels, however cluster 3 spent significantly more time watching tv. Cluster 1 (mix gender), cluster 4 (girls), cluster 6 (mix) and cluster 7 (boys) all represented medium parent-education level, while only one cluster (2; boys) represented high parent-education level. No discernable trend was evident for parental education level or gender with respect to sedentary time or behaviors. Cluster 2 had the highest PA level, followed by clusters 6 and 7, which is composed by students whose parents have medium or high educational level and greater levels of PA. As education level is considered a socioeconomic indicator [54], it could be considered that as parental educational level increases, so too does the higher purchasing power and time spent to carry out PA with their children. This fact could even reinforce the idea that educational level has an influence, when considering the socioeconomic context, as a result of the interactive effect of family- (i.e., family structure) and home-related (i.e., environment) factors [1]. Cluster 1 had significantly greater amounts of sedentary time and the most sedentary behaviors than all other clusters, however similar to the primary school group, this cluster represented a small minority (6.5%) of the sample. Surprisingly, cluster 2 with high education-parental level and majority boys, reported significantly greater levels of sedentary time and the most sedentary behaviors than all other cluster groups (except cluster 1). This finding is not in line with some other research, which has highlighted that both higher parental education level and boys are more likely to be related to greater levels of PA and reduced sedentary activities [12,30,37,55]. One explanation may be that by this age, children’s priorities shift towards educational pursuits such as homework, which is routinely more sedentary in nature. A number of studies have highlighted that, irrespective of socioeconomic status or parent-educational level, parents are regularly engaged with their child’s homework [56,57]. Whereas only high parent-education level is routinely associated with higher PA in children [5,30], Therefore, there may be a trade-off between children spending less amount of time engaging in physical activity, and instead higher amount of time pursing academic activities. One other consideration may be that the majority of this sample group came from a medium parent education level, and an appropriate sample size of high parent-education level may not have been achieved to draw accurate conclusions.

Interestingly, high school age children reported the fewest number of PA profile clusters. While cluster 1 represented boys and cluster 2 represented girls (both with a mix of parental education), no difference was reported between their PA level, sedentary time, or sedentary behaviors. It is likely that by this age, teenagers are more self-directed in their PA and sedentary behaviors, and are therefore less influenced by their parents’ education level [58]. With regards to gender, a number of studies have reported that teenagers have different reasons for physical inactivity, based upon their gender. Body image, self-efficacy and social pressures have been reported to play a role for females’ level of engagement in PA [59,60], while boys report high levels of self-determined motivation for MVPA and therefore their involvement in PA [61,62]. It is proposed that the promotion of healthy PA behaviors amongst teenagers should differ based upon the target demographic, such as gender and socioeconomic status [63]. Parents also require further education on the important benefits of PA for children of all ages.

The present study has some limitations that should to be accounted for in further research. First, the use of tracking systems and smart devices would allow to increase the PA information from children. Second, the psychological factors should be of interest when explaining the students’ characteristics (e.g., the role modeling or self-regulatory processes). This is a cross-sectional study and it informs about associations, but not about causal effects. Third, further investigations could increase the age and sample size of students to increase the reliability and generalizability of current findings.

## 5. Conclusions

The main findings of this study pointed out a number of behavioral profiles of school students accounting for key contextual factors such their age stage, sex, and parental education level. While these contextual factors have been previously associated with PA level and sedentary behavior individually, few studies have sought to examine them holistically by creating behavioral profiles. These behavioral profiles differed for students of different age stages. Higher parental education level was found to be particularly prevalent in behavioral profiles with greater levels of PA, for both primary and secondary students. This supports the growing body of work that has identified parental education as a factor in children’s PA level and sedentary behavior. Policy-makers charged with promoting healthy behaviors should consider targeted messages based on relevant contextual factors, such as age stage and parental education, to improve PA levels and reduce sedentary behaviors in school students.

## Figures and Tables

**Table 1:** Results of the age stage groups (clusters) identified by the two-step cluster analysis for infantile, primary, secondary and high school.

**Preschool**	**Cluster 1**		**Cluster 2**		**Cluster 3**		**Cluster 4**
*n*	46 (25.7%)		18 (10.1%)		49 (27.4%)		66 (36.9%)
Parents’ Educational Level	High (100%)		High (50%)		High (100%)		Medium (74.2%)
Time PA (h/week)	10.40 ± 10.28		8.63 ± 7.90		9.19 ± 6.98		8.50 ± 8.07
Sedentary Time (h/day)	5.89 ± 0.42		16.29 ± 4.70		6.60 ± 2.79		6.92 ± 2.55
Sex	Boys (100%)		Boys (66.7%)		Girls (100%)		Boys (53%)
Time watching TV (h/day)	3.69 ± 0.31		8.75 ± 3.75		4.25 ± 1.90		4.31 ± 1.95
Time using tablet/similar (h/day)	1.22 ± 0.22		3.51 ± 2.73		1.25 ± 1.23		1.25 ± 1.44
Time doing homework	0.97 ± 0.35		4.02 ± 8.86		1.09 ± 1.24		1.45 ± 2.54
**Primary**	**Cluster 1**	**Cluster 2**	**Cluster 3**		**Cluster 4**	**Cluster 5**	**Cluster 6**
*n*	60 (21.1%)	39 (13.7%)	74 (26.1%)		12 (4.2%)	45 (15.8%)	54 (19.0%)
Parents’ Educational Level	Low (100%)	High (100%)	Low (100%)		Medium (66.7%)	High (80.2%)	Medium (100%)
Time PA (h/week)	4.94 ± 6.14	10.19 ± 7.90	5.87 ± 6.43		6.99 ± 6.45	11.47 ± 10.44	10.02 ± 9.67
Sex	Boys (100%)	Boys (51.3%)	Girls (100%)		Boys (58.3%)	Girls (100%)	Girls (100%)
Sedentary Time (h/day)	8.75 ± 4.16	9.82 ± 4.81	8.54 ± 4.58		25.75 ± 2.00	7.60 ± 4.40	7.32 ± 4.46
Time using tablet/similar (h/day)	1.08 ± 1.96	2.20 ± 2.44	1.39 ± 2.35		8.75 ± 3.72	1.85 ± 2.55	1.47 ±1.96
Time watching TV (h/day)	5.36 ± 2.79	5.29 ± 3.17	5.22 ± 3.14		12.16 ± 3.35	3.80 ± 3.53	4.06 ± 3.50
Time doing homework (h/day)	2.30 ± 2.19	2.32 ± 1.07	1.91 ± 1.62		4.83 ± 1.02	1.94 ± 1.92	1.77 ± 1.67
**Secondary**	**Cluster 1**	**Cluster 2**	**Cluster 3**	**Cluster 4**	**Cluster 5**	**Cluster 6**	**Cluster 7**
*n*	15 (6.5%)	24 (10.4%)	59 (25.7%)	35 (15.2%)	34 (13.9%)	29 (12.6%)	34 (14.7%)
Parents’ Educational Level	Medium (100%)	High (100%)	Low (100%)	Medium (100%)	Low (100%)	Medium (60.8%)	Medium (100%)
Time PA (h/week)	7.95 ± 6.82	10.70 ± 8.93	4.01 ± 4.03	3.29 ± 2.72	6.25 ± 5.87	8.07 ± 7.61	9.71 ± 9.69
Sex	Boys (53.3%)	Boys (100%)	Girls (100%)	Girls (100%)	Girls (100%)	Boys (100%)	Boys (100%)
Sedentary Time (h/day)	33.64 ± 7.66	17.01 ± 8.26	13.11 ± 5.95	10.43 ± 6.65	13.17 ± 4.41	11.56 ± 5.45	11.67 ± 4.77
Time using tablet/similar (h/day)	15.75 ± 6.75	5.41 ± 3.54	3.97 ± 2.79	3.56 ± 3.17	4.14 ± 2.73	3.44 ± 2.57	4.17 ± 3.22
Time watching TV (h/day)	10.15 ± 3.91	5.47 ± 4.12	4.78 ± 3.55	3.26 ± 2.96	3.33 ± 1.85	4.24 ± 2.71	3.15 ± 2.03
Time doing homework (h/day)	7.74 ± 4.54	6.12 ± 5.41	4.35 ± 2.86	3.60 ± 3.19	5.69 ± 2.46	3.87 ± 2.80	4.33 ± 2.72
**High School**	**Cluster 1**				**Cluster 2**		
*n*	18 (52.9%)				16 (47.1%)		
Sex	Boys (100%)				Girls (100%)		
Time PA (h/week)	5.95 ± 6.01				4.39 ± 4.13		
Time watching TV (h/day)	4.32 ± 2.93				3.53 ± 2.92		
Time doing homework (h/day)	6.22 ± 3.70				7.06 ± 2.40		
Time using tablet/similar (h/day)	4.66 ± 3.41				5.33 ± 3.51		
Parents’ Educational Level	High (44.4%)				Low (44.8%)		
Sedentary Time (h/day)	15.21 ± 5.41				15.93 ± 4.68		

**Table 2:** Differences in the variables (one-way ANOVA) in the different clusters at each educational level.

	F	*p*	Effect Size	Comparisons Groups
**Preschool**				
Total PA time (per week)	0.490	0.690	0.008	
Sedentary Time	63.963	0.000	0.523	2 vs. 1, 3, 4
Time watching TV	26.067	0.000	0.309	2 vs. 1, 3, 4
Time using tablet/similar	12.076	0.000	0.172	2 vs. 1, 3 4
Time doing homework	7.8867	0.000	0.119	2 vs. 1, 3 4
**Primary**				
Total PA time (per week)	3.711	0.003	0.063	1 vs. 2, 5, 6 2 vs. 3 3 vs. 5, 6
Sedentary Time	37.551	0.000	0.403	2 vs. 4, 5, 6 4 vs. 1, 2, 3, 5, 6
Time watching TV	14.355	0.000	0.205	1 vs. 4, 5, 6 2 vs. 4, 5 3 vs. 4, 5, 6 4 vs. 5, 6
Time using tablet/similar	22.984	0.000	0.292	1 vs. 2 4 vs. 1, 2, 3, 5, 6
Time doing homework	6.596	0.000	0.106	4 vs. 1, 2, 3, 5, 6
**Secondary**				
Total PA time (per week)	6.376	0.000	0.146	2 vs. 5 3 vs. 1, 2, 6, 7 4 vs. 1, 2, 5, 6, 7 5 vs. 7
Sedentary Time	31.024	0.000	0.455	1 vs. 2, 3, 4, 5, 6, 7 2 vs. 3, 4, 5, 6, 7 3 vs. 4
Time watching TV	11.403	0.000	0.235	1 vs. 2, 3, 4, 5, 6, 7 2 vs. 4, 5, 7 3 vs. 4, 5, 7
Time using tablet/similar	30.093	0.000	0.447	1 vs. 2, 3, 4, 5, 6, 7 2 vs. 4, 6
Time doing homework	4.485	0.000	0.108	1 vs. 3, 4, 5, 6, 7 2 vs. 3, 4, 6, 7 4 vs. 5 5 vs. 6
**High School**				
Total PA time (per week)	0.992	0.327	0.030	
Sedentary Time	0.170	0.683	0.005	
Time watching TV	0.607	0.442	0.019	
Time using tablet/similar	0.318	0.577	0.010	
Time doing homework	0.591	0.448	0.018	
